# Neuroinflammation alters GABAergic neurotransmission in hyperammonemia and hepatic encephalopathy, leading to motor incoordination. Mechanisms and therapeutic implications

**DOI:** 10.3389/fphar.2024.1358323

**Published:** 2024-03-15

**Authors:** Marta Llansola, Yaiza M. Arenas, María Sancho-Alonso, Gergana Mincheva, Andrea Palomares-Rodriguez, Magnus Doverskog, Paula Izquierdo-Altarejos, Vicente Felipo

**Affiliations:** ^1^ Laboratory of Neurobiology, Centro de Investigación Príncipe Felipe, Valencia, Spain; ^2^ Umecrine Cognition AB, Solna, Sweden

**Keywords:** GABA, neuroinflammation, motor incoordination, cognitive function, hyperammonemia, hepatic encephalopathy, golexanolone, bicuculline

## Abstract

Enhanced GABAergic neurotransmission contributes to impairment of motor coordination and gait and of cognitive function in different pathologies, including hyperammonemia and hepatic encephalopathy. Neuroinflammation is a main contributor to enhancement of GABAergic neurotransmission through increased activation of different pathways. For example, enhanced activation of the TNFα–TNFR1-NF-κB-glutaminase-GAT3 pathway and the TNFα-TNFR1-S1PR2-CCL2-BDNF-TrkB pathway in cerebellum of hyperammonemic rats enhances GABAergic neurotransmission. This is mediated by mechanisms affecting GABA synthesizing enzymes GAD67 and GAD65, total and extracellular GABA levels, membrane expression of GABA_A_ receptor subunits, of GABA transporters GAT1 and GAT three and of chloride co-transporters. Reducing neuroinflammation reverses these changes, normalizes GABAergic neurotransmission and restores motor coordination. There is an interplay between GABAergic neurotransmission and neuroinflammation, which modulate each other and altogether modulate motor coordination and cognitive function. In this way, neuroinflammation may be also reduced by reducing GABAergic neurotransmission, which may also improve cognitive and motor function in pathologies associated to neuroinflammation and enhanced GABAergic neurotransmission such as hyperammonemia, hepatic encephalopathy or Parkinson’s disease. This provides therapeutic targets that may be modulated to improve cognitive and motor function and other alterations such as fatigue in a wide range of pathologies. As a proof of concept it has been shown that antagonists of GABA_A_ receptors such as bicuculline reduces neuroinflammation and improves cognitive and motor function impairment in rat models of hyperammonemia and hepatic encephalopathy. Antagonists of GABA_A_ receptors are not ideal therapeutic tools because they can induce secondary effects. As a more effective treatment to reduce GABAergic neurotransmission new compounds modulating it by other mechanisms are being developed. Golexanolone reduces GABAergic neurotransmission by reducing the potentiation of GABA_A_ receptor activation by neurosteroids such as allopregnanolone. Golexanolone reduces neuroinflammation and GABAergic neurotransmission in animal models of hyperammonemia, hepatic encephalopathy and cholestasis and this is associated with improvement of fatigue, cognitive impairment and motor incoordination. This type of compounds may be useful therapeutic tools to improve cognitive and motor function in different pathologies associated with neuroinflammation and increased GABAergic neurotransmission.

## 1 Introduction

Hepatic encephalopathy (HE) is a neuropsychiatric syndrome in which cerebral function is impaired as a consequence of a previous liver failure. HE is present mainly in patients with liver cirrhosis. The symptoms of HE progresses through a wide range of cognitive and motor abnormalities, from mild cognitive impairment and motor incoordination to disorientation, sleep alterations, changes in personality, between other alterations. HE may lead to coma and death. Around 40%–60% of patients with liver cirrhosis present minimal HE (MHE), with psychomotor slowing, attention deficits, mild cognitive impairment and motor incoordination, which strongly reduce their ability to perform tasks of the daily life and increase the risk of accidents, falls and hospitalizations, reducing the life quality and span of the patients and posing an important economic burden to the health systems (Leevy et al., 2007; Bajaj et al., 2012; Stepanova et al., 2012; Felipo, 2013; Flamm et al., 2018; Häussinger, 2022). MHE affects several million people around the World and is a serious medical, social and economic problem. MHE is not evident, but may be detected using psychometric tests. MHE may progress to clinical HE, with evident symptoms, which may lead to coma and death (Felipo, 2013; Häussinger, 2022).

Currently there are no specific treatments for MHE. Only the use of rifaximin, a non-permeable antibiotic, has been approved to prevent the appearance of episodes of clinical HE (Bass et al., 2010). It is therefore necessary to develop new treatments to reverse the cognitive and motor alterations in patients with MHE. To design and test new treatments for MHE it is necessary to understand the molecular mechanisms by which liver injury leads to cognitive and motor impairment. Hyperammonemia and peripheral inflammation act synergistically to induce MHE (Shawcross et al., 2004; Felipo et al., 2012; Balzano et al., 2020a). The process leading to neurological impairment in MHE is being characterized and the underlying mechanisms are being identified in animal models of hyperammonemia and MHE. The general process by which liver injury induces MHE involves sequential induction of hyperammonemia and inflammation, which trigger neuroinflammation, leading to altered neurotransmission which finally impairs cognitive and motor function (see Cabrera-Pastor et al., 2019; Haüssinger et al., 2022; Balzano et al., 2023 for review and references therein). On the bases of the advances in the knowledge of the mechanisms by which liver injury induces MHE, several new treatments have been successfully tested to restore cognitive and motor function in animal models (Hernandez-Rabaza et al., 2015; Dadsetan et al., 2016a; Dadsetan et al., 2016b; Hernandez-Rabaza et al., 2016; Balzano et al., 2020b; Balzano et al., 2023).

The final inductor of the neurological impairment is the alteration of neurotransmission, mainly of GABAergic and glutamatergic neurotransmission (Cauli et al., 2006; Cauli et al., 2009a; Llansola et al., 2015). It has been proposed that altered GABAergic neurotransmission in cerebellum plays a relevant role in the pathogenesis of autism (Blatt, 2005), Huntington’s disease (Garret et al., 2018), Alzheimer’s disease (Luchetti et al., 2011; Assefa et al., 2018; Calvo-Flores Guzmán et al., 2018), Parkinson’s disease and multiple sclerosis (Luchetti et al., 2011) and in hyperammonemia and hepatic encephalopathy (Cauli et al., 2009b; Hassan et al., 2019; Arenas et al., 2022a; Arenas et al., 2022b).

The cerebellum is affected at early stages of HE, with altered cerebral blood flow, which correlates with impairment of performance in number connection test, bimanual coordination, visuo-motor coordination and in the Stroop test (Felipo et al., 2014). These functions are modulated by the cerebellum (Miall et al., 2001; Debaere et al., 2004; Pollock et al., 2007; Nabeyama et al., 2008; Segarra et al., 2008; Braga-Neto et al., 2012). Butz et al. (2010), also showed that ataxia, tremor and slowing of finger movements, functions also modulated by the cerebellum, are early markers of cerebral dysfunction at least in a subgroup of cirrhotic patients. Altered cerebellar function is therefore a main contributor to many early neurological alterations in cirrhotic patients.

## 2 Motor coordination is modulated by GABAergic neurotransmission in cerebellum

Motor coordination is mainly modulated in cerebellum by circuits modulating synaptic integration involving both GABA inhibition and NMDA receptor activation (Watanabe et al., 1998). Hanchar et al. (2005) showed that small increases in a tonic GABA conductance in cerebellar granule neurons account for the adverse effects of ethanol on motor coordination, supporting that enhanced GABAergic activation in cerebellum impairs motor coordination. The association between GABAergic neurotransmission and motor incoordination induced by ethanol is further supported by the report of Hood and Buck (2000) showing that there is an association between allelic variations in the GABA_**A**_ receptor γ2 subunit and the genetic susceptibility to ethanol-induced motor incoordination. Also, mutation of the inhibitory ethanol site in GABA_A_ ρ1 receptors promotes tolerance to ethanol-induced motor incoordination (Blednov et al., 2017).

Woo et al. (2018) showed that augmentation of tonic GABA release by astrocyte-specific overexpression of MAOB resulted in motor incoordination, with a reduced motor performance in the rotarod. They propose that astrocytic tonic GABA release can control motor coordination by tonically inhibiting cerebellar neuronal excitability and that there is a dynamic control of motor coordination via selective modulation of the release, synthesis, and clearance of astrocytic GABA. Chiu et al. (2005) showed that mice lacking the GABA transporter subtype 1 (GAT1) show increased levels of extracellular GABA and reduced motor coordination as assessed in the rotarod as well as tremor and ataxia. In the same line, the GAT1 inhibitor tiagabine, a clinically useful antiepileptic drug, also induces motor incoordination, tremor and ataxia (Salat et al., 2015; Bresnahan et al., 2019).

The above studies and other reports in the literature show that motor coordination is mainly modulated by GABAergic neurotransmission in cerebellum.

## 3 There are sex-dependent differences in motor coordination performance, in GABAergic neurotransmission and in their alterations in some pathological situations

There are sex-dependent differences both in motor coordination and in GABAergic neurotransmission under physiological conditions and also in the alterations induced by different pathological conditions in motor coordination and GABAergic neurotransmission.

These sex-dependent differences have been observed both in humans and in animal models.

In humans, young males have better bimanual coordination compared to their female counterparts (Shetty et al., 2014). Boys aged 6–9 years had higher scores than girls on eye-hand coordination (Canli et al., 2023).

There are also sex-dependent differences in the main proteins modulating GABAergic neurotransmission, including GABA_A_ receptors. Women have a higher GABA_A_-benzodiazepine receptors availability than men across all brain regions: frontal, parietal, anterior cingulate, temporal and occipital cortices, and cerebellum (Esterlis et al., 2013).

Human males show higher levels of the α1 subunit of GABA_A_ receptors while older females showed lower α2, α5, and β3 subunit expression in superior temporal gyrus (Pandya et al., 2019). The authors propose that these changes might significantly influence GABAergic neurotransmission and lead to sex-specific disease susceptibility and progression.

Alcohol intoxication depresses neural activity by enhancing inhibition mediated by GABAergic neurotransmission. Brain then compensates with a compensatory reduction of GABA. Marinkovic et al. (2022) analysed the effects of binge drinking of alcohol on GABA levels in brain as analysed by magnetic resonance and found that GABA levels were higher in light drinkers women compared to light drinkers men.

Sex-dependent differences in motor coordination and GABAergic neurotransmission have been also reported in animal models. A sex-dependent deficit in motor coordination has been also reported in the HdhQ200/200 mouse model of Huntington’s disease. These mice show a reduction in motor coordination at 8 and 10 months of age, with a more severe phenotype in female mice (Cao et al., 2021). Similar worse effects in females than in males have been reported in human patients with Huntington’s disease (Hentosh et al., 2021).

In rats, female substantia nigra reticulata (SNR) neurons contained more KCC2 compared with age-matched males and this is associated with sex-dependent differences in GABA_A_ receptor function, promoting the sexual differentiation of the SNR (Galanopoulou et al., 2003). Kirson et al. (2021) reported that GABAergic spontaneous inhibitory postsynaptic currents (sIPSC) kinetics the central amygdala (CeA) differ between females and males. Moreover, ethanol increases sIPSC frequency in males but not in females.

GABAergic neurotransmission and motor coordination are also affected differentially in males and females by environmental contaminants. Low-level developmental lead exposure in mice induces male-specific reduction of motor coordination in the rotarod (Leasure et al., 2008). Cauli et al. (2013) also showed sex differential effects of developmental exposure to polychlorinated biphenyl 126 on motor coordination. PCB 126 impaired motor coordination at 2 months in males but not in females. Motor coordination was impaired by developmental exposure to endosulfan, cypermethrin, and chlorpyrifos in female rats but not in males. The effect of endosulfan and cypermethrin would be due to increased extracellular GABA in cerebellum, which remains unaltered in male rats (Gomez-Gimenez et al., 2018). Prenatal exposure to chlorpyrifos increases the expression of GABA_A_ receptor α1 subunit in females (Biosca-Brull et al., 2023).

## 4 GABAergic neurotransmission is enhanced in cerebellum in hyperammonemia and hepatic encephalopathy

A role for enhanced GABAergic neurotransmission in the pathogenesis of acute hepatic encephalopathy was proposed around 40 years ago by Schafer and Jones (1982). Schafer et al. (1984) observed that the pattern of the visual evoked potentials in rabbits with acute hepatic coma was identical to that in comas induced by drugs which activate GABAergic neurotransmission. They investigated the possible mechanisms by which GABAergic neurotransmission would be enhanced in acute hepatic encephalopathy and proposed that it would be due to increased levels of endogenous benzodiazepine ligands (Jones et al., 1993). They later proposed that hyperammonemia also contributes to enhance GABAergic neurotransmission by increasing the levels of neurosteroids and enhancing the binding of benzodiazepines to GABAA receptor and potentiating GABA-induced chloride currents (Jones and Basile, 1997; Jones, 2002).

We have later shown that alterations in GABAergic neurotransmission play a key role in the induction of motor and of some cognitive alterations in hyperammonemia and MHE (see below). Cauli et al. (2009b) showed, by brain microdialysis in freely moving rats that GABAergic neurotransmission is enhanced in cerebellum *in vivo* in rats with chronic hyperammonemia and MHE, but is reduced in cerebral cortex of the same rats. Similar findings were reported later in humans for cirrhotic patients with hepatic encephalopathy as analysed by transcranial magnetic stimulation (TMS). Hassan et al. (2019) using TMS showed an enhanced GABAergic neurotransmission in cerebellum of cirrhotic patients with HE *in vivo*. Groiss et al. (2019), also by TMS, showed a reduction of GABAergic neurotransmission of the motor cortex *in vivo* in patients with HE. This enhanced GABAergic neurotransmission plays a key role in the impairment of motor function and coordination, as discussed below.

## 5 Neuroinflammation is responsible for enhanced GABAergic neurotransmission in cerebellum in hyperammonemia

The increase in GABAergic neurotransmission in cerebellum in hyperammonemia and MHE is a consequence of neuroinflammation. Hyperammonemia induces neuroinflammation in cerebellum, with activation of microglia and astrocytes, increased levels of TNFα and increased membrane expression of the TNFα receptor TNFR1 (Hernandez-Rabaza et al., 2016; Cabrera-Pastor et al., 2018; Balzano et al., 2020b). This results in enhanced activation of two signaling pathways which modulate GABAergic neurotransmission: the TNFα - TNFR1- NF-κB–glutaminase- GAT3 pathway and the TNFα - TNFR1- S1PR2 - CCL2 - BDNF - TrkB pathway.

### 5.1 TNFα-TNFR1-NF-κB–Glutaminase-GAT3 pathway

**FIGURE 1:**
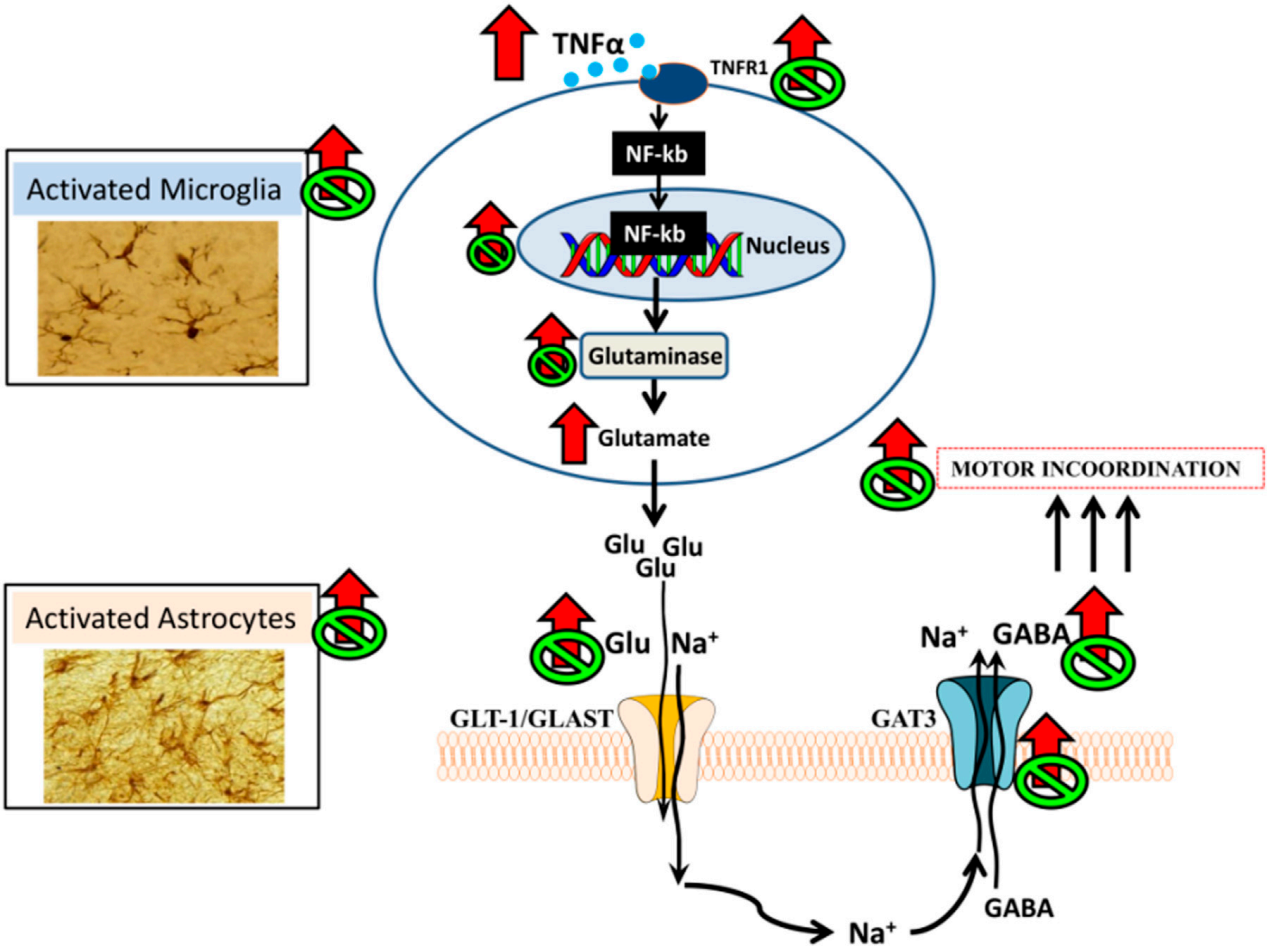
Proposed pathway by which neuroinflammation would increase extracellular GABA in cerebellum and impair motor coordination in hyperammonemic rats and proposed mechanism for its restoration by extracellular cGMP (Cabrera-Pastor et al., 2018).

Enhanced activation of TNFR1 leads to translocation to the nucleus of NF-κB in Purkinje neurons. This transcription factor induces an increase in the content of pro-inflammatory factors and of glutaminase, resulting in increased production of glutamate. This is associated with increased levels of extracellular glutamate in cerebellum of hyperammonemic rats *in vivo*, as assessed by microdialysis. The increase in extracellular glutamate leads to enhanced glutamate and Na^+^ uptake by glutamate transporters in activated astrocytes. The increased uptake of Na^+^ into astrocytes alters the transmembrane sodium gradient and leads to reversal of the function of the GABA transporter GAT3, which, instead of taking up GABA from the extracellular fluid into the astrocytes releases GABA from the astrocytes to the extracellular fluid, resulting in increased levels of extracellular GABA, as shown by *in vivo* brain microdialysis (Heja et al., 2009; Hernandez-Rabaza et al., 2016; Khakh et al., 2017; Cabrera-Pastor et al., 2018). The increase of extracellular GABA in cerebellum contributes to induce motor incoordination in hyperammonemic rats. Enhanced activation of this TNFα - TNFR1- NF-κB–glutaminase- GAT3 pathway, the increase in extracellular GABA and motor incoordination are reversed by administration of extracellular cGMP (Figure 1; Cabrera-Pastor et al., 2018).

### 5.2 TNFα-TNFR1-S1PR2-CCL2-BDNF-TrkB pathway

Enhanced activation of TNFR1 in cerebellum of hyperammonemic rats also leads to an increase in membrane expression of the S1PR2 (sphingosine-1-phosphate receptor 2) which induces an increase of CCL2 in Purkinje neurons. CCL2 is released to the extracellular fluid and activates its receptor CCR2 in microglia, leading to microglia activation and increased synthesis of BDNF. Microglia release BDNF which activates its receptor TrkB in Purkinje neurons (Arenas et al., 2022a). As summarized in the next section, the BDNF-TrkB system modulates GABAergic neurotransmission at different steps, by different mechanisms, and alterations in this modulation play a role in several pathologies. We therefore analyzed if enhanced levels of BDNF and TrkB activation contribute to enhanced GABAergic neurotransmission in cerebellum of hyperammonemic rats. We found that increased activation of TrkB leads to increased content of GABA-synthesizing enzymes GAD65 and GAD67, resulting in increased GABA synthesis that contributes to its increased extracellular concentration. Increased activation of TrkB also increases the membrane expression of the GABA transporter GAT3 in activated astrocytes, which also contributes to enhanced extracellular GABA concentration, as discussed above and to increased membrane expression of GABA_A_ receptors. All these effects contribute to enhanced GABAergic neurotransmission in cerebellum of hyperammonemic rats and are reversed by blocking TrkB with ANA12 (N-[2-[(2-oxoazepan-3-yl)carbamoyl]phenyl]-1-benzothiophene-2-carboxamide), supporting that they are mediated by the enhanced activation of TrkB by the increased BDNF levels (Arenas et al., 2022b).

**FIGURE 2:**
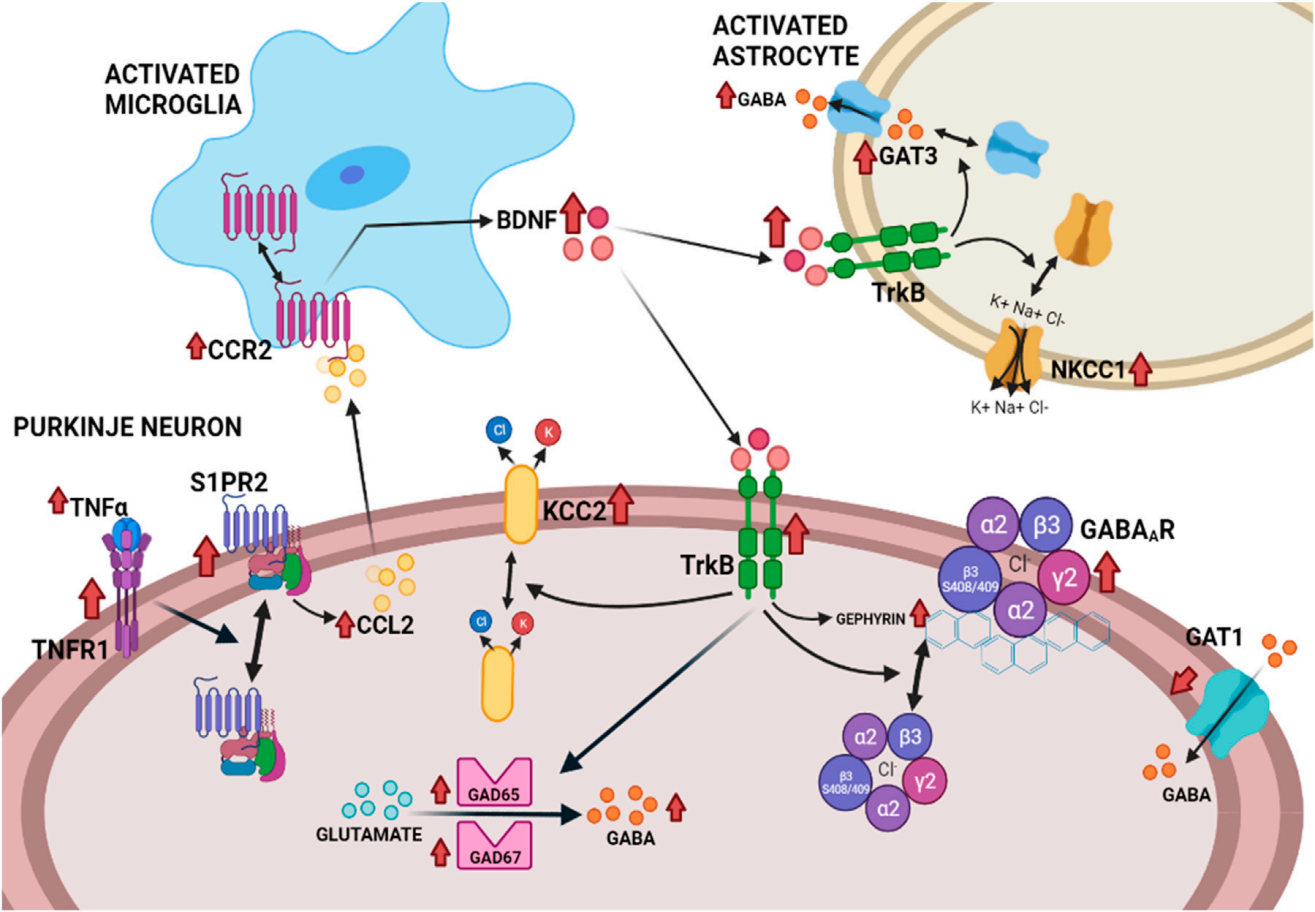
Proposed pathway by which the neuroinflammation-induced enhanced activation of the TNFR1–S1PR2–CCL2–CCR2–BDNF–TrkB pathway enhances GABAergic neurotransmission in the cerebellum of hyperammonemic rats (Arenas et al., 2022b).

The increase of CCL2 in Purkinje neurons and of BDNF in microglia are reversed by blocking S1PR2 with JTE-013 (1-[1,3-Dimethyl-4-(2-methylethyl)-1H-pyrazolo [3,4-b]pyridin-6-yl]-4-(2,6-dichloro-4-pyridinyl)-semicarbazid) indicating that enhanced activation of S1PR2 precedes these effects of hyperammonemia, while activation of TNFR1 by TNFα precedes over-activation of S1PR2, which is reversed by blocking TNFR1 signaling with R7050 (8-Chloro-4-(phenylsulfanyl)-1-(trifluoromethyl)[1,2,4]triazolo [4,3-a]quinoxaline, 8-Chloro-4-(phenylthio)-1-(trifluoromethyl)-[1,2,4]triazolo [4,3-a]quinoxaline) (Arenas et al., 2022a). The whole TNFα - TNFR1- S1PR2 - CCL2 - BDNF - TrkB–GABAergic neurotransmission pathway is summarized in Figure 2, from Arenas et al. (2022b). Increased GABAergic neurotransmission induced by enhanced activation of this pathway contributes to motor incoordination in hyperammonemic rats, which is reversed by blocking S1PR2 *in vivo* with the antagonist JTE-013, which reverses the increased activation of the S1PR2 - CCL2 - BDNF–TrkB pathway, normalizes GABAergic neurotransmission and reverses motor incoordination (Arenas et al., 2022a).

## 6 Modulation of GABAergic neurotransmission by the BDNF-TrkB system. Pathological implications

The above results indicate that hyperammonemia-induced neuroinflammation enhaces the activation of the BDNF-TrkB pathway. This pathway, in turn is a main modulator of GABAergic neurotransmission under physiological conditions. Moreover, alterations in the BDNF-TrkB system contribute to altered GABAergic neurotransmission in different pathological situations. This suggested that the altered function of the BDNF-TrkB pathway would also alter GABAergic neurotransmission in hyperammonemia and MHE.

It has been shown that the BDNF-TrkB system regulates GABAergic neurotransmission by different mechanisms, modulating expression (Bulleit et al., 2000; Roberts et al., 2006), phosphorylation (Vithlani et al., 2013) and trafficking (Brünig et al., 2001; Tomoda et al., 2022) of GABA_A_ receptors, GABA release (Bardoni et al., 2007; Vaz et al., 2015), GABA transporter GAT-1 (Vaz et al., 2011), the function of the potassium-chloride co-transporter 2 (KCC2), which fine-tune intracellular chloride and the intensity of GABA_A_ receptor currents (Dai et al., 2014) and the expression of the GABA-synthesizing enzyme GAD67 (Arenas et al., 1996).

The net effect of BDNF-TrkB on GABAergic neurotransmission may be different for different cell types. For example, BDNF exposure decreased GABA responses in cultured mouse cerebellar granule cells through TrkB receptor-mediated signalling. However, the same system potentiates GABA responses in Purkinje neurons (Cheng and Yeh, 2005). The effects may be also different at different developmental stages. For example, the action of BDNF on GABA_A_ receptor currents changes from potentiating to suppressing during maturation of rat hippocampal CA1 pyramidal neurons (Mizoguchi et al., 2003).

Alterations in the BDNF-TrkB-GABA system has been proposed to contribute to different pathologies such as depression (Guilloux et al., 2012), neuropathic pain (Lever et al., 2003), stress-induced hyperalgesia (Dai et al., 2014), insomnia (Feng et al., 2019), panic disorder (Casarotto et al., 2010), epilepsy (Roberts et al., 2006), and also modulates neurogenesis (Vithlani et al., 2013), morphine reward (Koo et al., 2014) and sensory neurons (Pezet et al., 2002).

## 7 Effects of enhanced activation of the BDNF-TrkB system on GABAergic neurotransmission in hyperammonemia and hepatic encephalopathy

Enhanced activation of TrkB by BDNF affects several aspects of GABAergic neurotransmission in cerebellum of hyperammonemic rats (see Figure 2), including: increased levels of GAD65 and GAD67, the main enzymes synthesizing GABA; increased levels of total and extracellular GABA; increased membrane expression of GAT3 in activated astrocytes, which contributes to the increase of extracellular GABA, as discussed above; increased levels of gephyrin and of phosphorylation of the β3 subunit of GABA_A_ receptors, two of the main modulators of trafficking of these receptors, which result in increased membrane expression of GABA_A_ receptors. Hyperammonemia also alters the membrane expression of the K^+^-Cl^-^ cotransporters KCC2 and NKCC1, that are key modulators of the transmembrane chloride gradient and, therefore, of the intensity of the responses to activation of GABA_A_ receptors (Arenas et al., 2022b).

All these effects are reversed *ex vivo* in cerebellar slices from hyperammonemic rats by blocking TrkB with ANA12, indicating that all the above effects of hyperammonemia on GABAergic neurotransmission are mediated by enhanced function of the BDNF-TrkB system (Arenas et al., 2022b). Many of the above changes occur mainly in Purkinje neurons, leading to enhanced GABAergic neurotransmission, in agreement with the enhanced GABAergic tone in Purkinje neurons reported by Hassan et al. (2019) in cirrhotic patients with hepatic encephalopathy.

## 8 Enhanced GABAergic neurotransmission is responsible for motor incoordination in hyperammonemia and hepatic encephalopathy

Enhanced GABAergic neurotransmission in cerebellum is responsible for motor incoordination in rat models of chronic hyperammonemia and hepatic encephalopathy.

A correlation between extracellular GABA levels in cerebellum *in vivo* and motor incoordination has been reported in rats with chronic hyperammonemia (Gonzalez-Usano et al., 2014). Moreover, reducing extracellular GABA by administration of pregnenolone sulfate restored motor coordination in hyperammonemic rats (Gonzalez-Usano et al., 2014).

Extracellular GABA in cerebellum of hyperammonemic rats may be also reduced by reducing neuroinflammation. Hernandez-Rabaza et al. (2016) showed that treatment with sulforaphane (1-Isothiocyanato-4-(methylsulfinyl)-butane) reduces neuroinflammation in cerebellum, leading to normalization of extracellular GABA and of motor coordination. Similar effects were obtained by treating the hyperammonemic rats with sildenafil (Agusti et al., 2017), by increasing extracellular cGMP (Cabrera-Pastor et al., 2018), by blocking peripheral TNFα with infliximab (Dadsetan et al., 2016b) or by blocking the S1PR2 with JTE-013 (Arenas et al., 2022b).

## 9 Reducing GABAergic neurotransmission reduces neuroinflammation and restores cognitive and motor function in rats with hyperammonemia and hepatic encephalopathy

We have proposed that there is an interplay between GABAergic neurotransmission and neuroinflammation, which regulate each other (Agusti et al., 2017) and contribute to modulation of motor coordination and cognitive function. This interplay implies that enhanced GABAergic neurotransmission in some pathological situations (i.e., hyperammonemia, hepatic encephalopathy, Parkinson’s disease) may be reduced by reducing neuroinflammation, as mentioned above. This interplay also implies that neuroinflammation may be reduced in similar pathological situations by reducing GABAergic neurotransmission. This has been demonstrated in rats with chronic hyperammonemia and hepatic encephalopathy treated with bicuculline, an antagonist of GABA_A_ receptors, or with golexanolone, a GABA_A_ receptor-modulating steroid antagonist that reduces GABAergic neurotransmission by reducing the potentiation of GABA_A_ receptor activation by neurosteroids such as allopregnanolone (Johansson et al., 2015).

### 9.1 Treatment with bicuculline improves neuroinflammation and cognitive and motor function in hyperammonemic rats

Cauli et al (2009b) showed by *in vivo* brain microdialysis in freely moving rats that chronic hyperammonemia increases GABAergic tone in cerebellum but decreased it in the cerebral cortex. They reported that increased GABAergic tone in the cerebellum of rats with hyperammonemia could have been caused by increases in extracellular GABA; tetrahydrodeoxy-corticosterone (a neurosteroid that enhances GABA_A_ receptor activation); or amounts of the alpha1, alpha6, and gamma2 subunits of GABA_A_ receptors. The decrease in GABAergic tone observed in the cortex could have resulted from the reduced amount of GABA_A_ receptors delta and gamma2 subunits or increased levels of pregnanolone (5-fold), which selectively reduces activation of GABA_A_ receptors that contain alpha4 subunits (widely expressed in cortex but not in cerebellum). Treatment with bicuculline normalized GABAergic tone and restored the function of the glutamate-nitric oxide-cGMP pathway in cerebellum *in vivo*. Moreover, treatment with bicuculline also restored learning ability of hyperammonemic rats in a Y maze conditional discrimination task, which is modulated by the function of the glutamate-nitric oxide-cGMP pathway (Erceg et al., 2005; Cabrera-Pastor et al., 2016).

The same effects are induced by treatment with pregnenolone sulphate, a negative allosteric modulator of the GABA_A_ receptor, which also improves the motor incoordination caused by increased extracellular GABA in the cerebellum (Gonzalez-Usano et al., 2014).

Malaguarnera et al. (2021) hypothesized that GABA_A_ receptors can modulate cerebellar neuroinflammation and that reducing GABAergic neurotransmission by chronic treatment with bicuculline would reduce neuroinflammation and improve motor coordination in cerebellum of hyperammonemic rats. They found that bicuculline reduced both peripheral inflammation and neuroinflammation in cerebellum. In hyperammonemic rats, bicuculline decreases IL-6 and TNFα and increases IL-10 in plasma: Moreover, bicuculline reduces astrocyte activation and induces the differentiation of microglia from a pro-inflammatory to an anti-inflammatory phenotye, and reduces IL-1β and TNFα in the cerebellum. Bicuculline restores the membrane expression of some glutamate and GABA transporters restoring the extracellular levels of GABA in hyperammonemic rats. This was associated with improvement of motor coordination in these rats.

Malaguarnera et al. (2021) showed that blocking GABA_A_ receptors improves peripheral inflammation and cerebellar neuroinflammation, restoring neurotransmission in hyperammonemic rats, whereas it induces inflammation and neuroinflammation in controls. This suggests a complex interaction between GABAergic and immune systems. The modulation of GABA_A_ receptors could be a suitable target for improving neuroinflammation and motor coordination in hyperammonemia, hepatic encephalopathy and other pathologies such as Parkinson’s disease.

Similar beneficial effects of bicuculline on neuroinflammation in hippocampus were also reported by Malaguarnera et al. (2019). Treatment with bicuculline reduces astrocyte activation and IL-1β but not microglia activation in the hippocampus of hyperammonemic rats. Bicuculline reverses the changes in membrane expression of AMPA receptor subunits GluA1 and GluA2 and of the NR2B (but not NR1 and NR2A) subunit of NMDA receptors. Bicuculline improves spatial learning and working memory and decreases anxiety in hyperammonemic rats. They proposed that in hyperammonemia, enhanced activation of GABA_A_ receptors in the hippocampus contributes to some but not all aspects of neuroinflammation, to altered glutamatergic neurotransmission and to impairment of spatial learning and memory as well as anxiety, all of which are reversed by reducing activation of GABA_A_ receptors with bicuculline.

### 9.2 Treatment with golexanolone improves neuroinflammation and cognitive and motor function in rat models of hyperammonemia, hepatic encephalopathy and cholestatic liver disease

The above studies with bicuculline were a proof of concept that reduceing GABAergic neurotransmission reduces neuroinflammation and improves motor function in hyperammonemia and hepatic encephalopathy. However, antagonists of GABA_A_ receptors such as bicuculline are not the ideal therapeutic tools because they may also induce deleterious effects. Therefore, new therapeutic agents able to reduce GABAergic neurotransmission by other mechanisms with less secondary effects are being developed. One of these compounds is golexanolone. Golexanolone (GR3027) is a new compound that selectively antagonizes the enhanced activation of GABA_A_ receptors by neurosteroids such as allopregnanolone and 3α,21-dihydroxy-5α-pregnan-20-one (THDOC). As described in above sections, studies in animal models show that over-activation of GABA_A_ receptors is involved in cognitive and motor impairment in hyperammonemia and hepatic encephalopathy and that reducing this activation restores cognitive and motor function. Johansson et al. (2015) assessed whether golexanolone improves motor incoordination and cognitive function in two different rat models of minimal hepatic encephalopathy: rats with chronic hyperammonemia and rats with portacaval shunts (PCS). Golexanolone improved motor coordination and spatial learning and memory in the radial maze and the Morris water maze. They proposed that golexanolone may be useful to improve cognitive and motor function in cirrhotic patients with MHE.

As a pilot study to assess the utility of golexanolone to improve MHE in cirrhotic patients, Montagnese et al. (2021) studied the safety, pharmacokinetics and efficacy of golexanolone in patients with liver cirrhosis. They showed that golexanolone exhibited satisfactory safety and pharmacokinetics and was well tolerated and associated with improvement in cognitive performance. These results support the therapeutic potential of golexanolone.

To further characterize the beneficial effects of golexanolone on neurological function in hyperammonemia and hepatic encephalopathy, Mincheva et al. (2022) assessed if treatment of hyperammonemic rats with golexanolone reduces peripheral inflammation and neuroinflammation and restores cognitive and motor function and analysed some underlying mechanisms. They showed that treating hyperammonemic rats with golexanolone reversed changes in peripheral inflammation, normalizing TNFα and IL-10 levels in plasma. Golexanolone also reversed microglia and astrocytes activation in cerebellum and hippocampus and restored motor coordination and spatial and short-term memory. This was associated with reversal of the hyperammonemia-enhanced activation in cerebellum of the TNFR1-glutaminase-GAT3 and TNFR1-CCL2-TrkB-KCC2 pathways.

Arenas et al. (2023) extended these type of studies to rats with bile duct ligation (BDL), which are a model of both hepatic encephalopathy and of cholestatic liver disease. These rats show, in addition to cognitive and motor impairment other symptoms characteristic of cholestatic liver disease such as fatigue. Many patients with the chronic cholestatic liver disease primary biliary cholangitis (PBC) show fatigue and cognitive impairment that reduces their quality of life. Current PBC treatments do not improve symptomatic alterations such as fatigue or cognitive impairment and new, more effective treatments are therefore required. Arenas et al. (2023) assessed the effects of golexanolone on fatigue and cognitive and motor function in cholestatic BDL rats as well as on peripheral inflammation, neuroinflammation, and GABAergic neurotransmission in the cerebellum. Golexanolone improves fatigue, memory impairment and motor incoordination and locomotor gait in BDL rats. This improvement was associated with reduction of neuroinflammation, with reversal of microglia activation and of astrocytes damage and reduction of pro-inflammatory IL-1β, TNFα, IL-6, and glutaminase and increase of anti-inflammatory IL-10 in the cerebellum. Golexanolone also reversed the increase in the GABA-synthesizing enzyme GAD67, the reduction in the GABA transporter GAT1, and the increase in the GABA β3 subunit of GABA_A_ receptors, thus reducing enhanced GABAergic neurotransmission. Golexanolone also reduced plasma levels of TNFα, IL-6, IL-17, and IL-18. These results further support that reducing activation of GABA_A_ receptors with golexanolone reduces neuroinflammation, improves GABAergic neurotransmission and fatigue and cognitive and motor impairment in models of hepatic encephalopathy and cholestasis. Similar beneficial effects would be expected in patients with primary biliary cholangitis and in cirrhotic patients with MHE.
